# Spin-Wave Channeling in Magnetization-Graded Nanostrips

**DOI:** 10.3390/nano12162785

**Published:** 2022-08-14

**Authors:** Rodolfo A. Gallardo, Pablo Alvarado-Seguel, Felipe Brevis, Alejandro Roldán-Molina, Kilian Lenz, Jürgen Lindner, Pedro Landeros

**Affiliations:** 1 Departamento de Física, Universidad Técnica Federico Santa María, Avenida España 1680, Valparaíso 2390123, Chile; 2 Center for the Development of Nanoscience and Nanotechnology (CEDENNA), Santiago 9170124, Chile; 3 Departamento de Matemáticas, Universidad de Chile, Las Palmeras 3425, Ñuñoa, Santiago 7800003, Chile; 4 Universidad de Aysén, Calle Obispo Vielmo 62, Coyhaique 5952039, Chile; 5 Helmholtz-Zentrum Dresden-Rossendorf, Institute of Ion Beam Physics and Materials Research, Bautzner Landstr. 400, 01328 Dresden, Germany

**Keywords:** spin waves, ferromagnetic strip, channeling

## Abstract

Magnetization-graded ferromagnetic nanostrips are proposed as potential prospects to channel spin waves. Here, a controlled reduction of the saturation magnetization enables the localization of the propagating magnetic excitations in the same way that light is controlled in an optical fiber with a varying refraction index. The theoretical approach is based on the dynamic matrix method, where the magnetic nanostrip is divided into small sub-strips. The dipolar and exchange interactions between sub-strips have been considered to reproduce the spin-wave dynamics of the magnonic fiber. The transition from one strip to an infinite thin film is presented for the Damon-Eshbach geometry, where the nature of the spin-wave modes is discussed. An in-depth analysis of the spin-wave transport as a function of the saturation magnetization profile is provided. It is predicted that it is feasible to induce a remarkable channeling of the spin waves along the zones with a reduced saturation magnetization, even when such a reduction is tiny. The results are compared with micromagnetic simulations, where a good agreement is observed between both methods. The findings have relevance for envisioned future spin-wave-based magnonic devices operating at the nanometer scale.

## 1. Introduction

Spin waves (SWs) are collective magnetic excitations that present a strongly anisotropic dispersion, which has meaningful consequences since group velocity and phase velocity are generally not parallel. Due to the diverse interactions present in a magnetic nanostructure, SWs exhibit interesting properties such as caustic [1,2,3,4,5,6,7,8,9,10], channeling [11,12,13], and nonreciprocal characteristics [14,15,16,17,18,19,20,21,22,23,24,25,26,27,28,29,30,31]. The complexity of the band structure and the various ways of manipulating its behavior represent the main attraction for the scientific community in the field of magnonics, which utilizes propagating spin waves for nanoscale transmission and processing of information [32,33]. The interdisciplinary aspects of the broad field of magnonics are summarized in some excellent works that highlight the role of the SWs in different areas like magnon spintronics [34,35], spin caloritronics [36], magnonic logic circuits [37,38], metamaterials and magnonic crystals [39,40,41], spin textures [29,42,43], magnonic-phononic crystals [44,45], and three-dimensional and curvilinear magnonics [46,47].

Because spin waves are sensitive to the spin texture and the internal field landscape, they are prone to propagate in a conducted way. If the magnetic material presents a texture like a domain wall, Winter modes [11] are excited and channeled along its center since the wall acts as a local potential well for the spin waves [48,49,50,51,52]. Nonetheless, the channeling of spin waves may be compromised if the magnetic texture changes, rendering the domain-wall stability a critical problem. Another example is a magnetic system with one or two finite dimensions, for which the internal field is reduced at the edges. Consequently, edge modes are excited at low frequencies [53,54,55]. However, the edge modes may be challenging to detect due to the small number of precessing spins. These drawbacks can be overcome by proper material manipulation at the nanoscale. For instance, a gradual change in a magnetic property may be the key to steering spin waves without relying on unstable domain walls or low cross-section edge modes. Hence, the propagation of the waves can be confined to nanoscale channels, which turns out to be fundamental for envisioned magnonic devices, such as circulators, isolators, phase shifters, and logic devices [56,57,58,59,60,61,62].

Exploiting gradual changes in a given physical property is not a new topic. Indeed, inside a conventional step-index optical fiber, the index of refraction is controlled using different dielectric materials, e.g., a core with a high index covered with another material with a smaller index [63]. The cladding material with the lower refraction index allows light channeling due to the total internal reflection [64]. In graded-index optical fibers, the index is more noticeable at the center of the dielectric material, which helps to bend the light into the fiber axis [65,66]. In graded-index magnonics, the main idea is to manipulate the internal field landscape to create potential wells that allow for steering the spin waves [67,68]. Such SW control can be realized in several ways, for instance, by changing the applied field, the saturation magnetization, the exchange coupling parameter, the anisotropy constant, or changing the shape and geometry of the magnetic material. For finite nanomagnets, the inhomogeneous demagnetizing field creates the proper conditions to channel spin waves because it acts as a confining potential well, leading to spin-wave localization [69,70,71,72,73] and mode quantization [74,75]. On the other hand, it is also possible to change the already nonuniform internal field by introducing gradual changes in a magnetic property, e.g. the saturation magnetization [76,77]. This kind of graded-magnonic system has been achieved using compositionally graded ferrites, where the saturation magnetization changes across the film thickness [78]. Other graded-magnetic systems, usually referred to as exchange-spring media, have been fabricated, with graduation either in the anisotropy constant [79,80,81,82,83] or in the exchange coupling strength [84,85,86].

This paper analyses the propagation of spin waves in magnetization-graded ferromagnetic (FM) nanostrips, where a reduction of the saturation magnetization along the width is assumed. In this two-dimensional magnonic fiber, it is demonstrated that the magnetic graduation induces the propagation of guided spin-wave modes, mainly excited within the zones with a reduced saturation magnetization. Under a substantial reduction of saturation magnetization, the channelized waves are excited at frequencies lower than the edge modes, making the excitation of only such steered modes feasible. Besides, under the increase of the strips’ width, it is found that SWs are remarkably conducted along the zones with reduced magnetization, even when such graduation is tiny. Part of the results is compared with micromagnetic simulations, with an excellent agreement between both methods.

## 2. Results and Discussion

**Figure 1:**
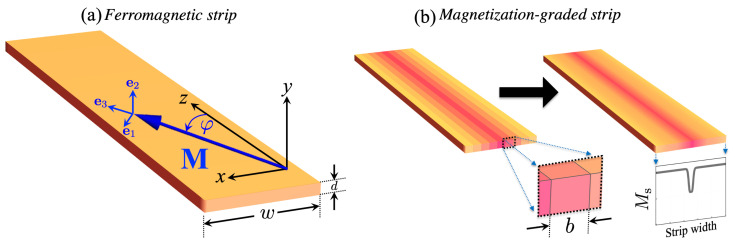
(**a**) Illustration of the coordinate system and the main geometrical parameters of a magnetic strip. In (**b**), a schematic representation of the dynamic matrix approach is shown, where the system is divided into many sub-strips of width *b*, which allows including magnetic graduation along the width. The color graduation represents the variation of the saturation magnetization along the width of the system.

The system under consideration is shown in Figure 1, where a thin magnetic nanostrip of width *w* and thickness *d* is illustrated. The magnetization M lies in the xz-plane and makes an angle φ measured from the *z*-axis. This section presents results for homogeneous ferromagnetic strips (with a constant saturation magnetization) and magnetization-graded strips. The detailed theoretical model for the stripe with nanoscale graduation of the magnetization is presented in the Appendices, where the dynamic matrix method is outlined in Appendix A together with the calculation of the matrix elements associated with the dipolar (Appendix B) and exchange (Appendix C) couplings. Standard values for Permalloy (Py: Ni${}_{80}$Fe${}_{20}$) will be used in the calculations. Namely, the saturation magnetization is $M_{s}=800$ kA/m, while the exchange constant is $A_{\mathrm{ex}}=9.9$ pJ/m. Also, the gyromagnetic ratio is γ=185.66 GHz/T, and the strip thickness is d=1 nm. It is worth mentioning that such a thickness is not a restriction in this work; similar results are obtained for d<10 nm. Regarding the discretization of the ferromagnetic strip, such a planar magnonic fiber is divided into small sub-strips, where b=4 nm is its width (see inset in Figure 1b). This size is less than the exchange length ($\ell_{\mathrm{ex}}=4.96$ nm) so that if $b<\ell_{\mathrm{ex}}$, the SW spectra do not noticeably change.

**Figure 2:**
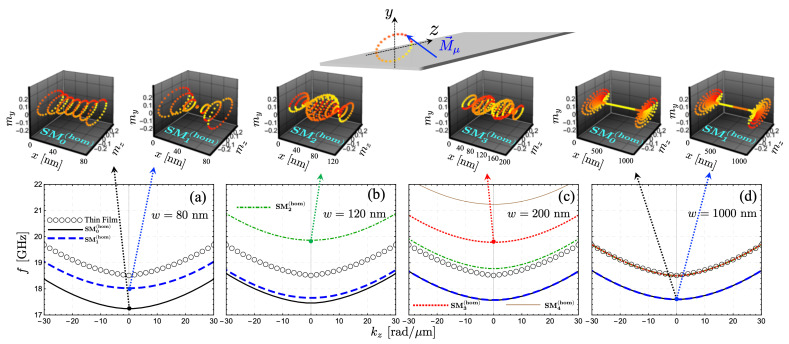
Spin-wave dispersion in a homogeneous ferromagnetic nanostrip. In (**a**–**d**), different values of the strip width, *w*, have been considered. The open circles show the SW dispersion of a ferromagnetic thin film, whereas the lines are the spin-wave modes of a magnetic nanostrip. Upper 3D graphics correspond to the spin-wave profiles along the strip’s width evaluated at $k_{z}=0$, where the dynamic magnetization components are calculated in arbitrary units. In all cases, SM${}_{0}^{\left(\mathrm{hom}\right)}$ and SM${}_{1}^{\left(\mathrm{hom}\right)}$ are edge modes, while other modes correspond to magnetic excitations with high SW amplitude around the strip center. In (**d**), the first five low-frequency modes have been calculated.

The transition from a nanostrip to an extended thin film is first studied to identify the nature of the calculated spin-wave modes characterized by the wave vector k. For this purpose, a bias field of $\mu_{0}H=$ 300 mT is applied along the *x* axis, while the spin waves propagate along *z*. The idea here is to study the Damon-Eshbach configuration (M⊥k), for which the modes will be notably influenced by the magnetic graduation, as shown below. Otherwise, in backward-volume configuration (M‖k), the modes are not significantly altered under a variation of the saturation magnetization (not shown). Therefore, in what follows, the equilibrium magnetization always points along *x* (φ=π/2), while the SW propagation is along the long axis *z*. Open circles in Figure 2a–d show the SW dispersion of an infinite thin film, while lines correspond to the modes of the FM nanostrip evaluated at different widths. By analyzing the spin-wave profiles across the width (upper 3D illustrations in Figure 2), it is observed that the spin excitations depicted in Figure 2a, for w=80 nm, correspond to edge modes since they are excited with high SW amplitudes at the strip borders. These modes are associated with a reduction of the internal field at the edges of the ribbon, wherein the surface magnetic charges generate a robust demagnetizing field at the edges. To establish a clear picture of the calculated spin-wave modes, they are labeled as SM${}_{\nu }^{\left(\mathrm{hom}\right)}$, with ν being the number of nodes and “hom” representing the homogeneous nanostrip case. Thus, SM${}_{0}^{\left(\mathrm{hom}\right)}$ and SM${}_{1}^{\left(\mathrm{hom}\right)}$ in Figure 2 correspond to edge modes of the homogeneous strip. Similar to the perpendicular standing spin waves in a thick FM film, the out-of-phase mode SM${}_{1}^{\left(\mathrm{hom}\right)}$ has higher dynamical energy than the in-phase one, SM${}_{0}^{\left(\mathrm{hom}\right)}$. As *w* increases, however, the edge modes become degenerate since the left and right borders are too far away from each other (see Figure 2c,d) so that the dynamic exchange energy is the same for both modes.

Higher-order modes are also observed at low frequencies, as depicted in Figure 2b–d. In Figure 2b, it is demonstrated that the mode SM${}_{2}^{\left(\mathrm{hom}\right)}$ is localized mainly in the center of the nanostructure, with two nodes at about 1/4 and 3/4 of the strip’s width. Otherwise, SM${}_{3}^{\left(\mathrm{hom}\right)}$ is excited with high SW amplitude around the strip center, with three nodes located about 1/6, 1/2 and 5/6 of the width. The other higher-order modes follow a typical distribution of a confined system with similar properties. The first five modes are displayed in Figure 2d for w=1000 nm, where one can observe that the bulk modes (localized around the nanostrip center) match with the one of an extended FM film. The degeneration of the modes shown in Figure 2d is anticipated since the mode quantization due to the geometrical confinement becomes irrelevant for large widths. Indeed, in a standard physical picture, the bulk SW modes in a ferromagnetic nanostrip exhibit different frequencies due to a term of the type $k_{⊥}=\nu \pi /w_{\mathrm{eff}}$ [87]. Here, $k_{⊥}$ is the quantized wave vector along the width, and $w_{\mathrm{eff}}$ is an effective strip’s width (proportional to *w*) that considers the dipolar boundary conditions at the strip’s edges. Thus, upon increasing *w*, the wave vector $k_{⊥}=\nu \pi /w_{\mathrm{eff}}$ tends to zero, and all standing waves are excited close to the ν=0 mode, which corresponds to the mode of an infinite film.

Once the nature of the spin waves in a ferromagnetic nanostrip is understood, the following step is to alter the magnetic properties along the strip’s width to induce spin-wave channeling. Note that the edge modes already provide a scenario for localized propagation due to a locally reduced internal field. Nonetheless, creating a sample with perfect edges is not a simple task. Such boundaries typically present an unavoidable roughness or defects that can prevent the natural behavior of the spin waves traveling in such zones. Another option is using domain walls that can channel the spin waves [12,13]. However, domain walls strongly depend on the material features and may be unstable against external magnetic fields. Besides, due to the nature of the formation of the walls, it is not easy to modify its shape, for instance, to bend the wall in arbitrary directions. Therefore, alternative ways to conduct the SWs are of high interest. In this context, magnetic graduation is proposed as a potential alternative for inducing a channelized propagation of spin waves at the nanoscale. Because the spin-wave dynamics strongly depend on the saturation magnetization, a local variation of $M_{s}$ along the nanostrip width will be considered. Of course, spin-wave steering is expected along the zone with reduced saturation magnetization since the magnetic moments experience a local low frequency [88], while the rest of the ferromagnetic strip is excited at a higher frequency. Nevertheless, a quantitative analysis is required because it is necessary to understand how large the magnetic graduation has to be chosen to create a notable SW channeling in the planar magnonic fiber shown in Figure 1b. Besides, it is essential to predict if a channelized mode can be the one with the lowest frequency so that only such mode is excited in a given range of frequencies. If the channelized mode is not the low-frequency one, it will be inevitable to excite it together with other modes, which could be undesirable for magnonic applications.

**Figure 3:**
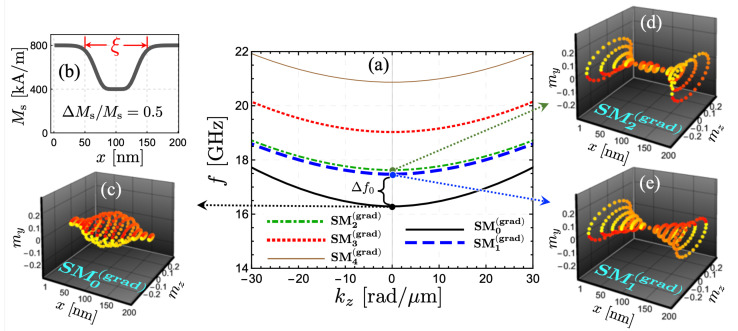
(**a**) Spin-wave dispersion of a magnetization-graded strip. The magnetic profile is shown in (**b**), where a notable reduction of $M_{s}$ along the width is assumed ($\Delta M_{s}/M_{s}=0.5$) and ξ=100 nm. (**c**, (**d**) and (**e**) depict the SW orbits along the strip width for modes SM${}_{0}^{\left(\mathrm{grad}\right)}$, SM${}_{1}^{\left(\mathrm{grad}\right)}$ and SM${}_{2}^{\left(\mathrm{grad}\right)}$, respectively. The dynamic magnetization components $m_{z}$ and $m_{y}$ are calculated with arbitrary units.

Figure 3 shows the case where the saturation magnetization has been varied along the strip’s width (for w=200 nm), with an $M_{s}$ reduction in the central region (see Figure 3b). Such a decrease in saturation magnetization is quantified by utilizing the parameters $\Delta M_{s}/M_{s}$ and ξ. Here, $\Delta M_{s}$ corresponds to the difference between its maximum and minimum values, and ξ measures the extension of the graduated zone. In the example shown in Figure 3, the fractional reduction is $\Delta M_{s}/M_{s}=0.5$ (50%) and ξ=100 nm. Overall, the SW band structure is notably modified, as shown in Figure 3a. An important aspect is that now the coherent mode, SM${}_{0}^{\left(\mathrm{grad}\right)}$ (where “grad” denotes the magnetization-graded stripe), is localized mainly in the center of the nanoribbon, where $M_{s}$ is reduced (see Figure 3c). The dynamic behavior of the low-frequency mode can be understood from the dependence of the SW frequency on saturation magnetization. In a typical Damon-Eshbach configuration, the frequency of the spin waves is reduced as $M_{s}$ decreases. Therefore, it is expected that in a magnetization-graded nanostructure, the lowest-frequency branch is excited in the zones where the saturation magnetization is reduced since such a zone is energetically compatible with low-frequency magnetic excitations. Of course, spin waves localized in zones with high $M_{s}$ are also feasible, but they are excited at high frequencies, as becomes evident by Figure 3. Besides, modes SM${}_{1}^{\left(\mathrm{grad}\right)}$ and SM${}_{2}^{\left(\mathrm{grad}\right)}$ are now the edge modes, which are not degenerate in frequency, as shown in Figure 3d,e. The highlighted frequency range, $\Delta f_{0}$, shown in Figure 3a, illustrates the frequencies at which only the mode SM${}_{0}^{\left(\mathrm{grad}\right)}$ can be excited. At more significant frequencies, it will be unavoidable to excite the channelized mode together with the higher-order ones. Note that width of transition from $M_{s}=800$ kA/m to 400 kA/m is about 25 nm. In the case of a more abrupt change of the saturation magnetization, even for a step-like change, the SW dispersion and the respective SW profiles do not significantly change (not shown). On the other side, the reduction of the saturation magnetization could be accompanied by the variation of other magnetic parameters, such as the gyromagnetic ratio. If the gyromagnetic ratio is reduced (increased) at the graduated zone, the channelized waves will slightly decrease (increase) their frequency.

**Figure 4:**
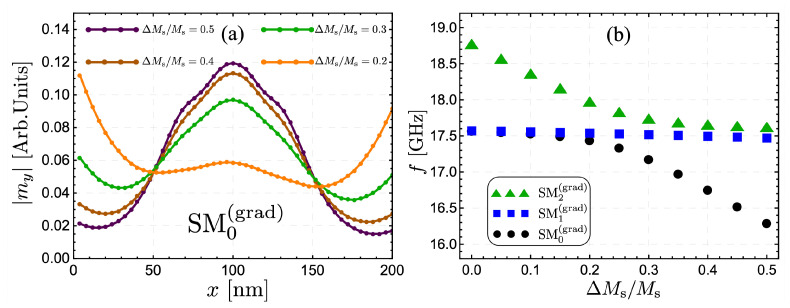
(**a**) The absolute value of the out-of-plane dynamic magnetization component, for mode SM${}_{0}^{\left(\mathrm{grad}\right)}$, as a function of *x* is shown for w=200 nm and ξ=100 nm. Different values of the fractional reduction of the saturation magnetization, $\Delta M_{s}/M_{s}$, have been accounted for. (**b**) The frequency of the modes SM${}_{\nu }^{\left(\mathrm{grad}\right)}$ (with ν=0, 1 and 2) is illustrated as a function of $\Delta M_{s}/M_{s}$ for $k_{z}=0$.

The reduction to 50% of the saturation magnetization ($\Delta M_{s}/M_{s}=0.5$) is a significant change in the magnetic graduation of the system. Therefore, it is interesting to explore where the SW excitations are concentrated as $\Delta M_{s}/M_{s}$ is changed. Figure 4a shows the absolute value of the out-of-plane component of the dynamic magnetization for mode SM${}_{0}^{\left(\mathrm{grad}\right)}$ as a function of *x*, the coordinate along the strip width, and evaluated at $k_{z}=0$ and ξ=100 nm. Here, it is possible to see how the SW localization evolves as graduation diminishes. Under a reduction by 30% ($\Delta M_{s}/M_{s}=0.3$), the mode SM${}_{0}^{\left(\mathrm{grad}\right)}$ is still mainly localized within the FM strip center. Nevertheless, when the reduction is only 20% (or less), the magnetization excitation is no longer localized in the strip center since the system tends to the homogeneous case, where the low-frequency mode is located at the strip edges. Also, the case $\Delta M_{s}/M_{s}=0.2$ depicts a more asymmetric profile along the strip’s width (see the orange curve in Figure 4a), which is owed to the hybridization between the modes SM${}_{0}^{\left(\mathrm{grad}\right)}$ and SM${}_{2}^{\left(\mathrm{grad}\right)}$. The dynamic behavior of the low-frequency mode, SM${}_{0}^{\left(\mathrm{grad}\right)}$, can be understood by analyzing Figure 4b, where the frequency of modes SM${}_{0}^{\left(\mathrm{grad}\right)}$, SM${}_{1}^{\left(\mathrm{grad}\right)}$, and SM${}_{2}^{\left(\mathrm{grad}\right)}$ are calculated as a function of $\Delta M_{s}/M_{s}$ at $k_{z}=0$. As the magnetic graduation increases, mode SM${}_{2}^{\left(\mathrm{grad}\right)}$ reduces its frequency (for $\Delta M_{s}/M_{s}<$ 0.3) since this dynamical state is mainly located at the zone where the saturation magnetization decreases. When the frequency of SM${}_{2}^{\left(\mathrm{grad}\right)}$ approaches the frequency range of edge modes (around 17.8 GHz), there is a coupling between SM${}_{0}^{\left(\mathrm{grad}\right)}$ and SM${}_{2}^{\left(\mathrm{grad}\right)}$ (see triangles and circles in Figure 4b). This coupling is responsible for SM${}_{0}^{\left(\mathrm{grad}\right)}$ increasing its localization at the nanostrip center, hence reducing its dynamic energy (or frequency). In contrast, SM${}_{2}^{\left(\mathrm{grad}\right)}$ takes over the role of an edge mode, as shown by the triangles in Figure 4b. It is worth noting that SM${}_{1}^{\left(\mathrm{grad}\right)}$ does not change its frequency notoriously because this mode has a node at the strip center and thus is only slightly influenced by the magnetic graduation.

**Figure 5:**
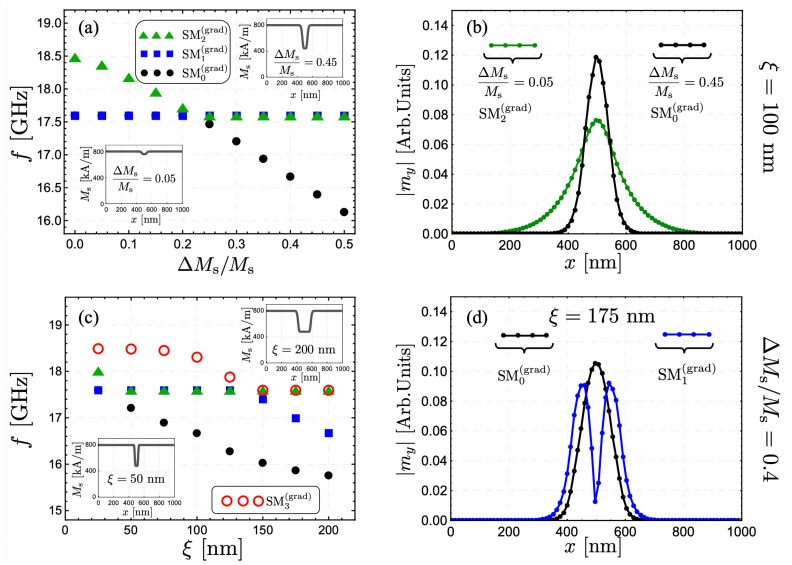
(**a**) Modes SM${}_{\nu }^{\left(\mathrm{grad}\right)}$ (with ν=0, 1 and 2) as a function of $\Delta M_{s}/M_{s}$. The modes are evaluated at $k_{z}=0$ and ξ=100 nm, for a wider strip with w=1000 nm. The crossing between modes reveals the weak coupling caused by the larger strip width *w*. The absolute value of the normal magnetization component for $\Delta M_{s}/M_{s}=0.05$ and 0.45 is illustrated in (**b**) for modes SM${}_{2}^{\left(\mathrm{grad}\right)}$ and SM${}_{0}^{\left(\mathrm{grad}\right)}$, respectively. (**c**) Modes SM${}_{\nu }^{\left(\mathrm{grad}\right)}$ (with ν=0, 1, 2 and 3) as a function of ξ. The case $k_{z}=0$, $\Delta M_{s}/M_{s}=0.4$, and w=1000 nm is considered. The magnetization profiles of modes SM${}_{0}^{\left(\mathrm{grad}\right)}$ and SM${}_{1}^{\left(\mathrm{grad}\right)}$ are shown in (**d**) for ξ=175 nm.

The coupling between the low-frequency mode and SM${}_{2}^{\left(\mathrm{grad}\right)}$ depends on the strip width since as *w* increases, the edge modes and SM${}_{2}^{\left(\mathrm{grad}\right)}$ (localized mainly at the strip center) are excited with SW amplitudes spatially far from each other. Therefore, exploring the channelized modes in the case of a wider ferromagnetic strip is also of interest. In Figure 5, a FM stripe with w=1000 nm is considered. In the case ξ=100 nm (see Figure 5a,b), it is evidenced that for the case $\Delta M_{s}/M_{s}<0.22$, the mode SM${}_{2}^{\left(\mathrm{grad}\right)}$ is channeled around the zone with reduced saturation magnetization, while at $\Delta M_{s}/M_{s}>0.22$ the mode SM${}_{0}^{\left(\mathrm{grad}\right)}$ becomes conducted along such zone. The crossing between modes in Figure 5a illustrates the weak coupling between the edge modes and SM${}_{2}^{\left(\mathrm{grad}\right)}$. A remarkable result is that an evident SW channeling is feasible even for the case $\Delta M_{s}/M_{s}=0.05$ (see the green curve in Figure 5b), which is not a trivial result since the graduation of 5% in the saturation magnetization is not a significant change. Besides, analogous to the previous case (w=200 nm), as $\Delta M_{s}$ increases, the channelized mode reduces its dynamical energy, becoming the low-frequency mode. It implies that it is attainable to excite only the channelized modes, which have a robust SW amplitude at the strip center, as shown in Figure 5b. Because of the strong localization of the low-frequency modes, it can be expected that the magnetization-graded zones can act as magnonic fibers or effective conduits for spin waves, similar to the SW propagation along domain walls [48,52]. Additionally, in the case of multiple channels, it is expected that the SWs can propagate with negligible interferences.

The evolution of the modes as a function of ξ is shown in Figure 5c, where the cases ν=0, 1, 2, and 3 are analyzed. Overall, one can observe that as ξ decreases, the low-frequency mode increases its dynamical energy. Such behavior is associated with the exchange energy that becomes high when the magnetic moments are forced to oscillate in a narrow zone. On the other hand, under a slight increase of ξ, the mode SM${}_{0}^{\left(\mathrm{grad}\right)}$ further decreases its frequency. Nonetheless, an additional mode with a node in the center of the graduated zone also reduces its frequency and, hence, is channeled (see blue SW profile in Figure 5d). This last effect is expected because as the graduated zone grows, the lateral standing waves excited within such area are energetically favorable at low frequencies.

**Figure 6:**
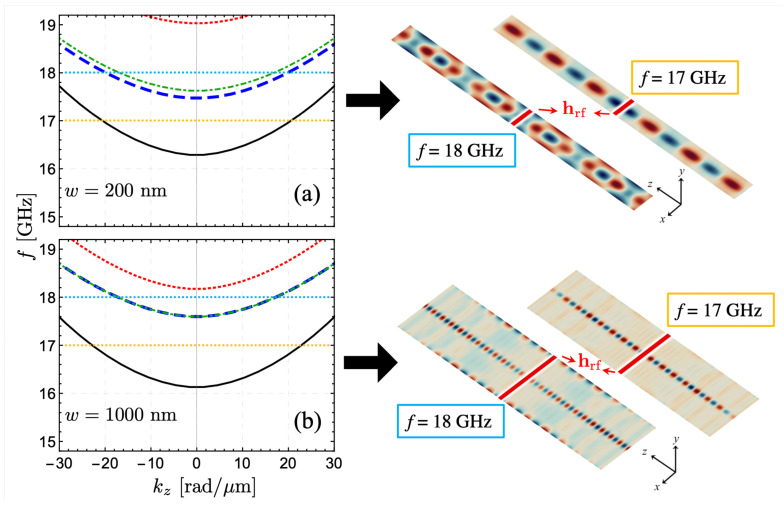
Calculated spin-wave dispersion and its respective profiles obtained from the micromagnetic simulations. In (**a**) and (**b**), the cases w=200 nm and w=1000 nm are respectively illustrated, where ξ=100 nm and $\Delta M_{s}/M_{s}=0.5$ are assumed in both cases. The different curves depict the low-frequency spin-wave modes. The SW propagation is simulated for f=17 GHz and 18 GHz (dotted horizontal lines). At f=17 GHz, the SWs are conducted along the nanostrip center, being this the unique excited mode, as the calculations predict. If the frequency of the field $h_{\mathrm{rf}}$ is 18 GHz, both the edge and the channelized modes are excited.

Finally, the calculated SW properties are compared with micromagnetic simulations based on the GPU-accelerated code MuMax3 [89]. Two different magnetic thin strips were considered with dimensions of 4096 nm ×200(1000) nm × 1 nm discretized into $2^{11}\times 2^{6}\left(2^{9}\right)\times 1$ cells along the (z,x,y) components, respectively, for two different widths; w=200 nm and w=1000 nm. Periodic boundary conditions along the *z*-direction were applied to simulate a long nanostrip. The graduation of $M_{s}$ was implemented by defining regions in the software with independent parameters. The nanostrip started with the magnetization along the x—direction due to an applied magnetic field of 300 mT (identical to the calculated cases) that saturates the sample. The generation of SWs was implemented through an external pulse in the form of $h_{\mathrm{rf}}=h_{0}\sin\left(2\pi f_{c}t\right)\hat{y}$, applied at the center of the sample over a width of 8 nm in *z*. Here, $f_{c}$ is the cut-off frequency, and $h_{0}$ was a hundred times smaller than the field used to saturate the sample. The system evolved for 5 ns. Figure 6 shows the calculated SW dispersions for the cases w=200 nm and w=1000 nm, where micromagnetic simulations are carried out at f=17 GHz and 18 GHz. For the smaller width, w=200 nm, the simulated SW propagation evidences the channeling of the waves at f=17 GHz, which is in concordance with the model’s prediction. Here, it is possible to observe that the edge modes are not excited at such a low frequency. Nevertheless, if the frequency of the field $h_{\mathrm{rf}}$ increases (18 GHz, for instance), the dynamic state of the system is compatible with the propagation of a channelized mode superimposed with the edge modes. This behavior is also seen for the wider sample (w=1000 nm), wherein the purely channelized mode is excited at a low frequency (17 GHz), while both the channelized and the edge modes are excited at a high frequency (18 GHz). These results demonstrate the validity of the model because it can predict the necessary conditions for spin waves steering along the nanostrip. Besides, under certain conditions, the profile of magnetic graduation chosen here allows to excite only the channeled modes since a notable reduction of the frequency is also reported.

The results presented here apply to ultrathin nanostructures, where the dynamic magnetization does not significantly vary across the nanostrip thickness. Therefore, it is required that $d\leq \ell_{\mathrm{ex}}$ so that at such thickness ranges, the exchange interaction is dominant, and the magnetic moments will oscillate parallel with each other along the strip thickness. Besides, if the graduation profile along the strip width changes, the localization of the magnetic excitations also changes. For instance, if the graduation is active just on one edge of the nanostructure, a robust localization of an edge mode will be induced in such a boundary (not shown). On the other hand, the experimental viability of creating graduated samples is currently feasible. For instance, the realization of vertical graduation along the thickness in thin films is achieved in epitaxial compositionally graded alloy films by co-sputtering, keeping the power of one material fixed while changing that of the other to achieve the intended composition profile [77,84,90]. Combinatorial material deposition can also be employed to fabricate samples with lateral gradients. A review of this method is found in Ref. [91], which typically results, however, in extended thin films. Further, lithographic masks can be used to manipulate the structures locally, allowing to achieve lateral gradients within single mesoscopic or nanoscopic structures. Some of the co-authors of this paper successfully used such a route on FeAl-alloys [92]. A direct way to locally modify magnetic properties employing a focused ion beam has been demonstrated in Refs. [93,94] and could be employed similarly to realize the graded nanostructures suggested in this paper.

## 3. Conclusions

The spin-wave spectra of magnetization-graded ferromagnetic nanostrips have been studied. A channelized spin-wave propagation has been predicted by modifying the saturation magnetization along the strip’s width. The spin-wave steering as a function of the fractional decrease of the saturation magnetization has been studied. It is demonstrated that spin waves can be conducted in magnetization-graded ferromagnetic strips, even when the saturation magnetization is slightly reduced. Besides, under the increase of the saturation magnetization contrast, the channelized spin-wave mode becomes the low-frequency one, allowing for exciting only such a mode. Micromagnetic simulations reveal the predicted channeled spin-wave propagation, validating the findings obtained with the theoretical calculations. These results are relevant for future applications associated with magnonic fibers and spin-wave steering at the nanometer scale.

## Data Availability

The datasets used and/or analyzed during the current study are available from the corresponding author on reasonable request.

## Appendix A. Dynamic Matrix Method

According to the system shown in Figure 1, an orthonormal basis $e_{1},e_{2},e_{3}$ is defined in relation to the basis $\hat{x},\hat{y},\hat{z}$ by a φ-rotation around the *y*-axis, so that $e_{3}$ points along the equilibrium configuration (see Figure 1a). Unless otherwise stated, spatial components are assumed to be relative to this basis, i.e., $\Lambda_{ij}=e_{i}\cdot \Lambda \cdot e_{j}$. Of course, it is expected that the magnetic moments at the lateral edges of the strip are canted due to an increase in the demagnetizing field, which is remarkable when the magnetization is along the width of the nanostructure (φ=±π/2). Nonetheless, in the current approach, it will be assumed that the external field $H_{Z}$ is strong enough to make the equilibrium magnetization fully parallel to it. To take into account the modulation of the dynamic magnetization along the strip’s width, the dynamic matrix method is used [95,96,97]. This method dictates the subdivision of the strip into *N* wire-shaped cells with small cross-sections of width *b* and thickness *d*, referred to as sub-strips, which are coupled by exchange and dipolar interactions. Then, by increasing the number *N* of subdivisions, the continuous system is described (see Figure 1b), which is validated through a convergence test.

Individual sub-strips have different magnetic properties, so the *n*-th cell possesses a magnetization $M^{n}\left(r,t\right)$. In this paper, Zeeman, exchange and dipolar interactions are considered, hence $H^{n}=H_{Z}+H_{\mathrm{ex}}^{n}+H_{\mathrm{dip}}^{n}$. The magnetization dynamics of each sub-strip is modeled by the Landau-Lifshitz equation ${\dot{M}}^{n}\left(r,t\right)=-\gamma M^{n}\left(r,t\right)\times H^{n}\left(r,t\right)$, for 1≤n≤N, with γ being the absolute value of the gyromagnetic ratio and the dot indicating a time derivative. Both magnetization and effective fields are written in terms of static (zeroth order) and dynamic (first-order) components, omitting higher-order terms, which enable linearization of the equations of motion. The dynamic components are assumed to behave like monochromatic plane waves, with the wave vector k confined to the *z*-axis. Thus $M^{n}\left(r,t\right)=M_{\mathrm{eq}}^{n}+m^{n}e^{i(k\cdot r-\omega t)}$ and $H^{n}\left(r,t\right)=H_{\mathrm{eq}}^{n}+h^{n}e^{i(k\cdot r-\omega t)}$, where $m^{n}$, $h^{n}\in C^{3}$ are complex vectors perpendicular to the equilibrium configuration ($M_{\mathrm{eq}}^{n}\left\|H_{\mathrm{eq}}^{n}\right\|H_{Z}$), and $M_{\mathrm{eq}}^{n}=M_{s}^{n}e_{3}$, with $M_{s}^{n}$ being the saturation magnetization. Also, the angular frequency is ω=2πf, where *f* is the frequency of the spin waves. By keeping terms up to first order in $m^{n}$ and $h^{n}$, the following system of linear equations is obtained,

(A1) $$\omega m^{n}=i\gamma \left(H_{\mathrm{eq}}^{n}\times m^{n}+h^{n}\times M_{\mathrm{eq}}^{n}\right).$$

A convenient linear structure is used for the dynamic effective field, namely, $h^{n}=-\sum_{p}\Lambda^{np}\cdot m^{p}$, where $\Lambda^{np}=\Lambda_{\mathrm{ex}}^{np}+\Lambda_{\mathrm{dip}}^{np}$, with $\Lambda_{\mathrm{ex}}^{np}$ and $\Lambda_{\mathrm{dip}}^{np}$ being the contribution of the exchange and dipolar couplings, respectively. Here $\Lambda_{\mathrm{dip}}^{np}$ plays the role of a dynamic multi-element demagnetizing tensor. With this in mind, the above system of linear equations becomes homogeneous. It can be interpreted as an eigenvalue problem of the form Tm=ωm, where $T\in L(C^{2N})$ is a linear operator acting on the vector $m=(m_{1}^{1},\ldots ,m_{1}^{N},m_{2}^{1},\ldots ,m_{2}^{N})$. Concerning the canonical basis of $C^{2N}$, the matrix representation of T is given by

(A2) $$T_{ql}=i\gamma \left({\left(-1\right)}^{j-1}H_{\mathrm{eq}}^{n}\delta_{np}\delta_{ij}+{\left(-1\right)}^{i-1}M_{\mathrm{eq}}^{n}\Lambda_{ij}^{np}\right),$$

where 1≤i,j≤2 and 1≤n,p≤N are integers uniquely defined by q=(2−i)N+n and l=(j−1)N+p, for each 1≤q,l≤2N. Here, $\delta_{ij}$ stands for the Kronecker delta symbol.

It is worth mentioning that analytical expressions for the tensor $T_{ql}$ are not found in the literature since the dynamic dipole-dipole interaction is not easy to handle in the case of a ferromagnetic nanostrip. For instance, expressions in terms of numerical integrations are given in Ref. [97] for the dynamic dipolar fields. In this manuscript, analytical expressions for the dipolar contribution are derived. Although somewhat extensive, these analytical terms allow obtaining the magnonic dispersion and magnetization profiles in a short time, representing an essential advantage over other numerical procedures or micromagnetic simulations. The detailed derivation of the dipolar and exchange contributions is presented in the following.

## Appendix B. Dipolar Interaction

The sub-strip geometry motivates the following definitions, which can be thought of as nothing more than a handy notation. The projection operator over the system’s bounded direction is $P=\hat{x}⊗\hat{x}+\hat{y}⊗\hat{y}$, and the axial radius is ρ=P·r. The operator $\int_{p}$ integrates a function over the *p*-th sub-strip cross section (b×d rectangle), and $\int_{R}$ denotes the integral over the real line along the long *z*-axis. Thus, one can note that $\int_{\Omega^{p}}=\int_{p}\int_{R}$. Expressions of the form $\int_{n}\int_{p}\eta \left(\rho -\rho^{\prime }\right)$ are considered, where the *p*-integral is over the primed coordinates, whereas the *n*-integral runs over the non-primed coordinates. Also, η is a well-behaved function with a singularity at the origin. More explicitly, it can be seen that

(A3) $$\int_{n}\int_{p}dS^{\prime }dS\;\eta \left(\rho -\rho^{\prime }\right)=\int_{\delta x^{np}}^{\delta x^{np}+b}\int_{\delta y^{np}}^{\delta y^{np}+d}\int_{0}^{b}\int_{0}^{d}dy^{\prime }dx^{\prime }dydx\;\eta \left(\rho -\rho^{\prime }\right),$$

for n≠p, and

(A4) $$\int_{n}\int_{n}dS^{\prime }dS\;\eta \left(\rho -\rho^{\prime }\right)=\lim_{\epsilon ,\delta \to 0^{+}}\int_{\delta }^{b-\delta }\int_{\delta }^{d-\delta }\int_{-\delta }^{b+\delta }\left(\int_{-\delta }^{y-\epsilon }+\int_{y+\epsilon }^{d+\delta }\right)dy^{\prime }dx^{\prime }dydx\;\eta \left(\rho -\rho^{\prime }\right),$$

for n=p. Here, dS=dxdy stands for the differential cross section area, and $\left(\delta x^{np},\delta y^{np}\right)$ represents the relative position of the *n*-th sub-strip with respect to the *p*-th sub-strip. At this point, introducing the shorthand notation $\int_{n}\int_{p}\eta$ may seem unnecessary. However, as the manuscript moves forward, its role of simplifying the discussion on otherwise tedious mathematical details will become evident. For example, it is interesting to consider the effect of changing the order of derivation and integration. If n≠p, the operators commute. However, if n=p, it follows from the above equation that

(A5) $$\int_{n}dS\left(\nabla \int_{p}-\int_{p}\nabla \right)dS^{\prime }\;\eta \left(\rho -\rho^{\prime }\right)=d\;\hat{y}\lim_{\epsilon ,\delta \to 0^{+}}\int_{\delta }^{b-\delta }\int_{-\delta }^{b+\delta }dx^{\prime }dx\;\eta \left(\rho -\rho^{\prime }\right){|}_{y^{\prime }=y+\epsilon }^{y-\epsilon }.$$

The magnetic field due to the *p*-th sub-strip is given by

(A6) $$H_{\mathrm{dip}}^{\left(p\right)}\left(r,t\right)=-\frac{1}{4\pi }\nabla \int_{p}\int_{R}dz^{\prime }dS^{\prime }\;M^{p}\left(r^{\prime },t\right)\cdot \nabla^{\prime }\left(\frac{1}{|r-r^{\prime }|}\right).$$

Then, the average magnetic field over the *n*-th sub-strip cross section due to the *p*-th sub-strip, $H_{\mathrm{dip}}^{np}=\left(\int_{n}H_{\mathrm{dip}}^{\left(p\right)}\right)/\left(bd\right)$, is considered. Note that the contribution to the effective field is given by $H_{\mathrm{dip}}^{n}=\sum_{p}H_{\mathrm{dip}}^{np}$. It is straightforward to write the dynamic component of the average field $H_{\mathrm{dip}}^{np}$ as $h_{\mathrm{dip}}^{np}=-\Lambda_{\mathrm{dip}}^{np}\cdot m^{p}$, where the dynamic multi-element demagnetizing tensor is given by

(A7) $$\begin{array}{cc} \Lambda_{\mathrm{dip}}^{np} & =\frac{e^{-ik\cdot r}}{4\pi bd}\int_{n}dS\;\nabla ⊗\int_{p}\int_{R}dz^{\prime }dS^{\prime }\;e^{ik\cdot r^{\prime }}\nabla^{\prime }\left(\frac{1}{|r-r^{\prime }|}\right) \end{array}$$

(A8) $$\begin{array}{c} =-\frac{1}{2\pi bd}\int_{n}dS\;\left(ik+\nabla \right)⊗\int_{p}dS^{\prime }\left(ik+\nabla \right)K_{0}(k|\rho -\rho^{\prime }\left|\right) \end{array}$$

(A9) $$\begin{array}{c} =\int_{n}\int_{p}dS^{\prime }dS\;K\left(\rho -\rho^{\prime }\right)+\delta_{np}\hat{y}⊗\hat{y}. \end{array}$$

Here the tensor field K is defined by

(A10) $$K\left(\rho \right)=\frac{1}{2\pi bd}\left(K_{0}\left(k\rho \right)k⊗k+i\frac{K_{1}\left(k\rho \right)}{\rho /k}\left(k⊗\rho +\rho ⊗k\right)\right.\left.+\frac{K_{1}\left(k\rho \right)}{\rho /k}P-\frac{K_{2}\left(k\rho \right)}{\rho^{2}/k^{2}}\rho ⊗\rho \right),$$

with $K_{l}$ denoting the *l*-th Bessel function of the second kind. Equation (A8) follows by noting that $\nabla |r-r^{\prime }|=-\nabla^{\prime }\left|r-r^{\prime }\right|$, commuting the operators $\int_{R}\nabla =\nabla \int_{R}$, using the identity $\int_{-\infty }^{\infty }e^{iaz}dz/\sqrt{z^{2}+b^{2}}=2K_{0}\left(|ab|\right)$ for a,b∈R, invoking the product rule $\nabla e^{ik\cdot r}\eta \left(\rho \right)=e^{ik\cdot r}\left(ik+\nabla \right)\eta \left(\rho \right)$ in succession, and canceling out the exponentials. Equation (A9) is obtained by considering the derivatives $K_{0}^{\prime }=-K_{1}$ and $K_{0}^{\prime \prime }=\left(K_{0}+K_{2}\right)/2$, the identity $2K_{1}\left(x\right)=x\left(K_{2}-K_{0}\right)\left(x\right)$, and using the commutation property shown in Equation (A5), where the resulting integrals are evaluated by taking $K_{1}\left(x\right)=1/x+O\left(x\right)$. Note that K is symmetric and traceless, so that the demagnetizing tensor $\Lambda_{\mathrm{dip}}^{np}$ is also symmetric, with trace 1 if n=p and trace 0 otherwise [97,98]. Also, $K_{xz}\left(x,y\right)=K_{yz}\left(y,x\right)$. Thus, in solving $\int_{n}\int_{p}K\left(\rho -\rho^{\prime }\right)$, only four components need to be considered: $K_{xx},K_{xy},K_{xz}$ and $K_{zz}$. Here, the spatial components are chosen concerning the $\hat{x},\hat{y},\hat{z}$ basis.

Assuming that there exists a tensor field J such that $K=\partial_{xx}\partial_{yy}J$, it follows from Equations (A3) and (A4) that

(A11) $$\int_{n}\int_{p}dS^{\prime }dS\;K\left(\rho -\rho^{\prime }\right)=\sum_{s,t=-1}^{1}\left(2-3|s|\right)\left(2-3|t|\right)\times J\left(\delta x^{np}+sb,\delta y^{np}+td\right),$$

for n≠p, and

(A12) $$\int_{n}\int_{n}dS^{\prime }dS\;K\left(\rho -\rho^{\prime }\right)=\lim_{\delta \to 0^{+}}\left(J\left(\rho -\rho^{\prime }\right){|}_{y^{\prime }=-\delta }^{d+\delta }{|}_{y=\delta }^{d-\delta }+d\lim_{\epsilon \to 0^{+}}\frac{\partial J}{\partial y}\left(\rho -\rho^{\prime }\right){|}_{y^{\prime }=y+\epsilon }^{y-\epsilon }\right){|}_{x^{\prime }=-\delta }^{b+\delta }{|}_{x=\delta }^{b-\delta },$$

for n=p. However, no elementary representation of J does exist because Bessel functions have not enough antiderivatives. On the other hand, it is possible to seek a solution in their series expansions. Known series expansions of $K_{0}$, $K_{1}$ and $K_{2}$, around z=0, are

(A13) $$\begin{array}{cc} K_{0}\left(z\right) & =-\alpha_{E}-\ln\left(\frac{z}{2}\right)+O{\left(z\right)}^{2}, \end{array}$$

(A14) $$\begin{array}{cc} K_{1}\left(z\right) & =\frac{1}{z}+\left(-\frac{1}{2}+\alpha_{E}+\ln\left(\frac{z}{2}\right)\right)\frac{z}{2}+O{\left(z\right)}^{2}, \end{array}$$

(A15) $$\begin{array}{cc} K_{2}\left(z\right) & =\frac{2}{z^{2}}-\frac{1}{2}+O{\left(z\right)}^{2}, \end{array}$$

where $\alpha_{E}$ stands for the Euler-Mascheroni constant (≈0.577). Hence, to first order, K is determined by

(A16) $$\begin{array}{cc} K_{xx}\left(x,y\right) & =\frac{1}{2\pi bd}\left[\frac{y^{2}-x^{2}}{{\left(x^{2}+y^{2}\right)}^{2}}+\frac{k^{2}}{4}\frac{x^{2}-y^{2}}{x^{2}+y^{2}}\right]-\frac{K_{zz}\left(x,y\right)}{2}, \end{array}$$

(A17) $$\begin{array}{cc} K_{xy}\left(x,y\right) & =\frac{1}{2\pi bd}\left(\frac{k^{2}}{2}\frac{xy}{x^{2}+y^{2}}-\frac{2xy}{{\left(x^{2}+y^{2}\right)}^{2}}\right), \end{array}$$

(A18) $$\begin{array}{cc} K_{xz}\left(x,y\right) & =\frac{ik_{z}}{2\pi bd}\left(\frac{x}{x^{2}+y^{2}}-\frac{k^{2}}{4}x\right)-\frac{ik_{z}}{2}xK_{zz}\left(x,y\right), \end{array}$$

(A19) $$\begin{array}{cc} K_{zz}\left(x,y\right) & =-\frac{k^{2}}{2\pi bd}\left[\alpha_{E}+\ln\left(\frac{k}{2}\right)+\frac{1}{2}\ln\left(x^{2}+y^{2}\right)\right], \end{array}$$

with J given by

(A20) $$\begin{array}{cc} J_{xx}\left(x,y\right) & =\frac{1}{2\pi bd}\left(1+\frac{k^{2}}{2}\frac{x^{2}+y^{2}}{6}\right)\left[xy\arctan\left(\frac{y}{x}\right)+\frac{y^{2}-x^{2}}{4}\ln\left(x^{2}+y^{2}\right)\right]-\frac{1}{2}J_{zz}\left(x,y\right), \end{array}$$

(A21) $$\begin{array}{cc} J_{xy}\left(x,y\right) & =\frac{1}{2\pi bd}\left[\left(\frac{x^{2}-y^{2}}{2}+\frac{k^{2}}{2}\frac{x^{4}-y^{4}}{12}\right)\arctan\left(\frac{y}{x}\right)+\left(\frac{xy}{2}+\frac{k^{2}}{2}\frac{x^{3}y+xy^{3}}{12}\right)\ln\left(x^{2}+y^{2}\right)\right], \end{array}$$

(A22) $$\begin{array}{cc} J_{xz}\left(x,y\right) & =\frac{ik_{z}}{2\pi bd}\left[\frac{3x^{2}y-y^{3}}{6}\arctan\left(\frac{y}{x}\right)+\frac{3xy^{2}-x^{3}}{12}\ln\left(x^{2}+y^{2}\right)-\frac{k^{2}}{2}\frac{x^{3}y^{2}}{24}\right]-\frac{ik_{z}}{2}xJ_{zz}\left(x,y\right), \end{array}$$

(A23) $$\begin{array}{cc} J_{zz}\left(x,y\right) & =-\frac{k^{2}}{2\pi bd}\left[\left(\alpha_{E}-\frac{25}{12}+\ln\left(\frac{k}{2}\right)\right)\frac{x^{2}y^{2}}{4}+\frac{x^{3}y-xy^{3}}{6}\arctan\left(\frac{y}{x}\right)\right.\\ \; & +\left.\frac{6x^{2}y^{2}-x^{4}-y^{4}}{48}\ln\left(x^{2}+y^{2}\right)\right]. \end{array}$$

For n≠p, explicit formulas for the demagnetizing tensor follow immediately by Equation (A11). The self-interaction case (n=p) requires a little more computational effort. Using Equation (A12), the self-interaction integrals ${\;\int \int \;}_{n}K$ are calculated for each component, where it is obtained that

(A24) $$\begin{array}{c} \int_{n}\int_{n}dS^{\prime }dS\;K_{xx}\left(\rho -\rho^{\prime }\right)=\frac{1}{\pi }\left[2\arctan\frac{d}{b}+\frac{b}{d}\ln b-\frac{d}{b}\ln d+\frac{1}{2}\left(\frac{d}{b}-\frac{b}{d}\right)\ln\left(b^{2}+d^{2}\right)\right]\\ +\frac{bdk^{2}}{48\pi }\left[-25+12\alpha_{E}+12\ln\frac{k}{2}+8\frac{b}{d}\arctan\frac{d}{b}+6\frac{b^{2}}{d^{2}}\ln b\right.\left.-2\frac{d^{2}}{b^{2}}\ln d\right.\\ +\left.\left(6+\frac{d^{2}}{b^{2}}-3\frac{b^{2}}{d^{2}}\right)\ln\left(b^{2}+d^{2}\right)\right], \end{array}$$

(A25) $$\begin{array}{c} \int_{n}\int_{n}dS^{\prime }dS\;K_{zz}\left(\rho -\rho^{\prime }\right)=-\frac{bdk^{2}}{24\pi }\left[-25+12\alpha_{E}+12\ln\frac{k}{2}\right.\left.+8\left(\frac{b}{d}-\frac{d}{b}\right)\arctan\frac{d}{b}\right.\\ \left.+2\frac{b^{2}}{d^{2}}\ln b+2\frac{d^{2}}{b^{2}}\ln d+\left(6-\frac{b^{2}}{d^{2}}-\frac{d^{2}}{b^{2}}\right)\ln\left(b^{2}+d^{2}\right)\right], \end{array}$$

and that ${\;\int \int \;}_{n}K_{xy}$ and ${\;\int \int \;}_{n}K_{xz}$ vanish. It is worth mentioning that the second term in Equation (A12) vanishes for all components.

This approximation used before holds as long as the relative distance between two sub-strips falls below a certain threshold. In the case of self-interaction (n=p), the threshold is expected to surpass the characteristic length of the sub-strips, namely, the diagonal $\sqrt{b^{2}+d^{2}}$. The threshold is defined as the value *z* at which the relative error of any of the $K_{0}\left(z\right)$, $K_{1}\left(z\right)$, or $K_{2}\left(z\right)$ series expansions surpasses 0.05, which happens first for $K_{0}\left(z\right)$ at around z=1/3. Note that the argument of the Bessel functions is $z=k|\rho -\rho^{\prime }|$. Thus, in the wave-vector regime $k\leq 30\;\mathrm{rad}\;\mu m^{-1}$, the approximation holds for sub-strips at a relative distance of no more than 11 nm. For relative distances greater than 11 nm, Bessel functions can be evaluated at the center of the sub-strip. Otherwise, a complete treatment of the static dipolar interaction can be found in Ref. [97].

## Appendix C. Exchange Interaction

The exchange interaction is accounted for using two terms. One of them is associated to the exchange interaction within each sub-strip, which is written as $h_{\mathrm{ex}1}^{n}=-\ell_{\mathrm{ex}}^{2}k^{2}m^{n}$, where $\ell_{\mathrm{ex}}=\sqrt{2A_{\mathrm{ex}}/\mu_{0}M_{s}^{2}}$ is the exchange length. The other is related to the exchange interaction between neighboring sub-strips. In this case, the exchange energy density between two neighboring sub-nanostructures (*n* and n+1) is $\epsilon_{\mathrm{ex}2}=JM^{n}\cdot M^{n+1}/\left(M_{s}^{n}M_{s}^{n+1}\right)$. Then, by using $H_{\mathrm{ex}2}^{n}=-\left(\delta \epsilon_{\mathrm{ex}2}/\mu_{0}\delta M^{n}\right)/b$, the associated fields are

(A26) $$h_{\mathrm{ex}2}^{n}=\frac{J/b}{\mu_{0}M_{s}^{n}}\sum_{p}\frac{m^{p}}{M_{s}^{p}}\left(\delta_{n,p+1}+\delta_{n,p-1}\right),$$

and

(A27) $$H_{\mathrm{eq}-\mathrm{ex}2}^{n}=\frac{J/b}{\mu_{0}M_{s}^{n}}\sum_{p}\left(\delta_{p,n+1}+\delta_{p,n-1}\right)e_{3},$$

where $H_{\mathrm{eq}-\mathrm{ex}2}^{n}$ corresponds to the static exchange field. Thus, by using $h_{\mathrm{ex}}^{np}=-\Lambda_{\mathrm{ex}}^{np}\cdot m^{p}$, the tensor $\Lambda_{\mathrm{ex}}^{np}$ is given by

(A28) $$\Lambda_{\mathrm{ex}}^{np}=\ell_{\mathrm{ex}}^{2}k^{2}\delta_{np}-\frac{J}{b}\frac{1}{\mu_{0}M_{s}^{n}M_{s}^{p}}\left(\delta_{n,p+1}+\delta_{n,p-1}\right).$$

Finally, if the system is divided into many sub-strips, the continuous approach establishes that $J=2A_{\mathrm{ex}}/b$, where $A_{\mathrm{ex}}$ is the exchange constant of the magnetic material defined in the continuum limit.
